# How a Mycoparasite Employs G-Protein Signaling: Using the Example of *Trichoderma*

**DOI:** 10.1155/2010/123126

**Published:** 2010-09-08

**Authors:** Markus Omann, Susanne Zeilinger

**Affiliations:** Research Area of Gene Technology and Applied Biochemistry, Working Group Molecular Biochemistry of Fungi, Institute for Chemical Engineering, Vienna University of Technology, Getreidemarkt 9, 1060 Vienna, Austria

## Abstract

Mycoparasitic *Trichoderma* spp. act as potent biocontrol agents against a number of plant pathogenic fungi, whereupon the mycoparasitic attack includes host recognition followed by infection structure formation and secretion of lytic enzymes and antifungal metabolites leading to the host's death. Host-derived signals are suggested to be recognized by receptors located on the mycoparasite's cell surface eliciting an internal signal transduction cascade which results in the transcription of mycoparasitism-relevant genes. Heterotrimeric G proteins of fungi transmit signals originating from G-protein-coupled receptors mainly to the cAMP and the MAP kinase pathways resulting in regulation of downstream effectors. Components of the G-protein signaling machinery such as G*α* subunits and G-protein-coupled receptors were recently shown to play crucial roles in *Trichoderma* mycoparasitism as they govern processes such as the production of extracellular cell wall lytic enzymes, the secretion of antifungal metabolites, and the formation of infection structures.

## 1. Introduction

All living organisms are confronted with a plethora of different stimuli due to exposure to the environment. Recognition of these stimuli and appropriate cellular responses like induction of gene transcription and protein phosphorylation are crucial for survival. Sensed at the cells surface these signals are mediated to intracellular elicitors by transmembrane signaling pathways. Fungi in particular emit sex-specific pheromones to attract potential mating partners of the opposite mating type. Surviving of pathogenic and mycoparasitic fungi depends on host-derived signals allowing them to recognize their hosts [1, 2]. 

 For more than 70 years, species of the filamentous fungus *Trichoderma* have been known to be able to attack and metabolize plant pathogenic fungi, and therefore they are used as biocontrol agents [3]. Currently *Trichoderma*-based biological pesticides (e.g., SoilGard, Trichodex) are applied against a variety of plant pathogenic fungi like *Rhizoctonia solani*, *Botrytis cinerea*, *Sclerotium rolfsii*, *Sclerotinia sclerotiorum*, and *Fusarium* spp. [4–6]. 

Biocontrol is defined as a number of different mechanisms working synergistically to achieve disease control revealing complex interactions between biological control agents, plant pathogen, and plant [7]. These mechanisms could be either indirect like competition for nutrients and space, antibiosis and stimulation of plant-defense mechanisms, or direct like mycoparasitism [2]. 

*Trichoderma* spp. exhibit the ability to survive under unfavorable conditions predominating in ecological niches like salt marshes. Strains used in biological control should stand a wide range of temperatures, salinity, low moisture and show resistance to fungicides and chemicals used in soil treatment. These characteristics, together with their ability to produce highly efficient siderophores which chelate iron resulting in growth inhibition of other fungi make *Trichoderma* potent competitors [8–10]. For stimulation of plant-defense mechanisms *Trichoderma* produces proteins and low-molecular-weight compounds which prevent the plant from further infections [11]. Furthermore, *Trichoderma* secretes diverse secondary metabolites like pyrones, peptaibols, and terpenes, which can inhibit growth of plant pathogenic fungi [6]. 

Fungal mycoparasitism, the direct attack of one fungus on another, implies different processes occurring consecutively. These processes include recognition of the host, formation of morphological changes such as coiling around the host's hyphae and development of appressorium-like structures and subsequent penetration and killing of the host [2, 12, 13]. For penetration of the host's cell wall *Trichoderma* produces hydrolytic enzymes like chitinases, glucanases, and proteases [14]. To some extent production of these enzymes is already induced prior to physical contact with the host due to inducing diffusible host-derived factors [15, 16]. In addition, complementary molecules present at the surface of both the host and the mycoparasite can mediate physical contact [17]. The plant pathogen *R. solani* was shown to possess glycoproteins (lectins) on its surface which are able to agglutinate carbohydrate moieties present on *Trichoderma* hyphae [12] and thus trigger coiling of the mycoparasite around the host hyphae [4, 18]. 

For the activation of the mycoparasitic response, a model of different signaling pathways responding to multiple signals from the host can be assumed. This is based on findings that on the one hand lectins induce morphological changes like coiling around the host hyphae and appressorium development in *Trichoderma* even though they are ineffective inducers of the chitinolytic enzyme system. On the other hand cell wall degradation products are powerful inducers of chitinase production whereas they do not efficiently induce coiling. Furthermore lectins induce coiling only upon physical contact while parts of the chitinolytic enzyme system are already induced before direct contact between *Trichoderma* and its host. 

Receptor molecules, located within the mycoparasite's cell membrane, are supposed to be the linkage between these host-derived signals and intracellular signalling pathways of *Trichoderma* resulting in, for example, the activation of mycoparasitism-relevant genes. Recently, examination of these intracellular signaling pathways of *Trichoderma* began and revealed that G-protein signaling plays an important role in mycoparasitism [19–23].

## 2. G-Protein Signaling

### 2.1. Components of G-Protein Signaling in Fungi

Classical G-proteins are heterotrimers composed of three subunits termed G*α*, G*β*, and G*γ*, which are highly conserved from fungi to humans. In fungi, heterotrimeric G proteins play essential roles in sexual and pathogenic development, in secondary metabolism, in pheromone signaling cascades and processes determining fungal virulence [1, 24].

Fungal G*α* subunits can be divided into three major subgroups according to a phylogenetic tree generated by multiple alignment of fungal G-protein sequences. Subgroup I G*α* proteins are homologs of the mammalian G*α*_i_ subunit as both contain a consensus sequence for myristoylation (MGXXXS) at the N-terminus [25] and a site for ADP-ribosylation by pertussis toxin (CAAX) at the C-terminus [26]. G*α* proteins of subgroup I lower the intracellular cAMP level by inhibiting adenylyl cyclase [27]. Among subgroup II members, protein sequences are not as well conserved as of members of groups I or III [28]. Their functions are less obvious, and their direct effectors still remain to be identified [24]. Members of subgroup III posses a myristoylation site at the N-terminus and positively influence the intracellular cAMP level. In analogy to the mammalian G*α*_s_ family, members of subgroup III have been designated as adenylyl cyclase stimulating fungal G*α*_s_ subunits [1].

Most fungal species possess one representative of each G*α* subgroup. However, screening the whole genome of *Saccharomyces cerevisiae* revealed that *GPA1* and *GPA2* are the only two G*α* subunit-encoding genes in this organism and they cannot be placed unambiguously into one of the three subgroups described above. GPA1 shows sequence relationship to subgroup I but lacks the consensus site for pertussis toxin-dependent ribosylation [1, 29].

By screening the genomes of *Ustilago maydis* and *Aspergillus oryzae*, a fourth G*α* subunit has been identified in these fungi. Both, Gpa4 of *U. maydis* and GaoC of *A. oryzae*, exhibit some unusual features and therefore do not belong to one of the three subgroups described above. Phylogenetic analysis indicated that Gpa4 and GaoC are distinct thus excluding the presence of a conserved fourth class of G*α* subunits in fungi [30, 31].

Fungal G*β*-encoding genes were shown to affect the sexual and asexual life cycle of these organisms [32]. In addition, examination of G*β* in the model filamentous fungus *Neurospora crassa* suggested that this subunit is also essential for the complex formation and stability of G*α* and G*γ* [33]. G*γ* subunits form a large family of small proteins from which the majority of filamentous fungi possess only a single conserved member [32]. G*γ* deletion in *N. crassa* for instance led to the same phenotype as deletion of the G*β* subunit such as increased conidiation, female-sterility, and decreased intracellular cAMP levels, and in addition an altered concentration of the three G*α* proteins [33].

In fungi, G-protein signaling pathways elicit cellular responses like mating, cell division, growth, morphogenesis, and pathogenic development [34] but up to now only little information is available on the characteristics and functions of fungal G-protein-coupled receptors (GPCRs). Numerous fungal genomes are sequenced nowadays and comparative genomics resulted in the classification of fungal GPCRs intofor example, nine classes [31]: classes I and II include pheromone receptors related to *S. cerevisiae* Ste2p and Ste3p receptors; classes III and V consist of putative carbon source and cAMP sensors; class IV contains *Schizosaccharomyces pombe* Stm1p-like nitrogen sensors; class VI comprises a unique class in filamentous fungi representing GPCRs with an RGS domain in the cytoplasmatic moiety of the protein; members of classes VII and VIII share similarities with the rat growth hormone releasing factor (class VII) and the steroid receptor mPR (class VIII); and class IX contains fungal opsins similar to the bacterial retinal-binding rhodopsin with the well-characterized representatives NOP-1 and ORP-2 of *N. crassa* [35]. Subsequent to the release of the genome of the rice blast fungus *Magnaporthe grisea*, a large novel class of fungal GPCRs related to PTH11, a receptor required for the development of the appressorium, was defined [36, 37] and recently Zheng et al. [38] reported on the identification of three novel classes, each of which comprises one member in the plant pathogen* Verticillium* spp. with high sequence similarity to GPCRs of higher eukaryotes.

### 2.2. Regulation of G-Protein Signaling in Fungi

Activation/deactivation and the intensity of G-protein signaling are regulated by interactions of the G*α* subunit with GPCRs, G*βγ* subunits, GTPase-activating, and multiple other proteins [39, 40]. 

GTPase-activating proteins, such as RGS (regulator of G-protein signaling) proteins, act to accelerate hydrolysis of GTP to GDP on G*α* subunits and thereby terminate the transduced signal [39, 41]. While in *S. cerevisiae* the RGS protein Sst2 was found to control mating responses by promoting the hydrolysis of GTP on the G*α* subunit Gpa1 via binding to the Ste2 pheromone receptor [42], Rgs2, the second RGS protein of yeast, negatively regulates the Gpa2 G*α* subunit and glucose signaling via the Gpr1 GPCR [43]. The filamentous model fungus *Aspergillus nidulans* contains four RGS proteins (in addition to the RGS domain-containing GPCR GprK; [31]) among which FlbA and RgsA were shown to negatively regulate the subgroup I and III G*α* subunits FadA and GanB, respectively [44, 45]. In pathogenic fungi such as *Cryphonectria parasitica, M. grisea, Cryptococcus neoformans,* and *Metarhizium anisopliae*, RGS proteins were described to regulate G*α*-mediated signaling of fungal virulence [46–50]. 

Modulation of the activity of G*βγ* subunits can be achieved by proteins belonging to the family of phosducins or phosducin-like proteins. Reports on the function of these proteins in filamentous fungi are rare. In *A. nidulans* and the chestnut blight fungus *C. parasitica*, the phosducin-like proteins PhnA and BDM-1, respectively, were shown to be necessary for G*β* function [51, 52]. In addition, BDM-1 was recently reported to be a phosphoprotein and it was shown to play a positive role in regulation of virulence [53]. 

In addition to regulatory proteins influencing G-protein activity, mechanisms directly regulating the activation and stability of GPCRs exist. Although, there are not yet any reports on their mode of action in filamentous fungi, regulation of the Ste2 and Ste3 pheromone receptors has been studied in detail in the model organism *S. cerevisiae*. GPCR signaling was shown to be regulated by ligand-triggered Ste2 receptor oligomerization and phosphorylation resulting in receptor desentization, endocytosis, and internalization [54–56]. Like Ste2, Ste3 can be recycled via ligand dependent manner [57]. In addition, the Afr1 protein was found to prevent G-protein activation via the Ste2 receptor independent of receptor phosphorylation and endocytosis [58] whereas the Asg7 protein inhibits signaling by G*βγ* via a concerted action with the Ste3 pheromone receptor [59].

## 3. The Role of G-Protein Signaling in *Trichoderma* Mycoparasitism

Comparable to fungal pathogens which attack plant, animal or human hosts, mycoparasites are pathogenic to other fungi. The mycoparasitic attack involves similar processes as those described for other pathogenic fungi such as infection-related morphogenesis, the production of hydrolytic enzymes involved in host invasion, and mycotoxin synthesis. 

In plant pathogenic fungi, subgroup I and III G*α* proteins and the cAMP pathway were repeatedly shown to play an essential role in regulating virulence-associated processes such as filamentation and appressorium formation. Similarly, G-protein signaling also governs pathogenesis and the production of virulence factors in various fungal human pathogens such as *C. neoformans* and *Aspergillus fumigatus* (reviewed in [24]). 

**Figure 1:**
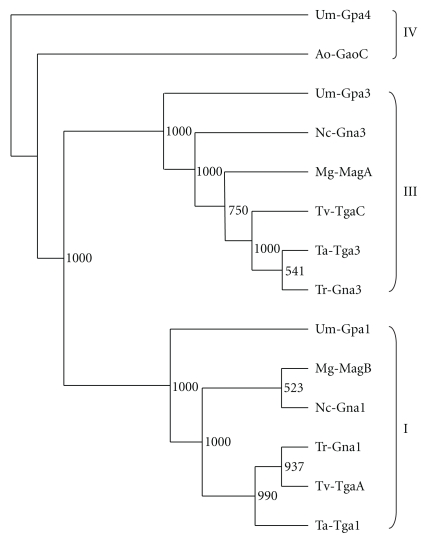
Relationship of fungal G*α* amino acid sequences of subgroups I and III whose members Tga1 and Tga3 were shown to be essential for mycoparasitism of *T. atroviride*. In addition, the fourth G*α* subunit only present in *U. maydis* (Gpa4) and *A. oryzae* (GaoC) were included and are indicated as subgroup IV. The tree was generated using Neighbor-Joining algorithm subsequent to CLUSTALX alignment. The fourth G*α* subunit of *U. maydis* Gpa4 has been defined as outgroup. Abbreviations used: Ao: *Aspergillus oryzae*; Mg: *Magnaporthe grisea*; Nc: *Neurospora crassa*; Ta: *Trichoderma atroviride*; Tr: *Trichoderma reesei*; Tv: *Trichoderma virens*; Um: *Ustilago maydis*.

Investigating different *Trichoderma* spp. for G-protein signaling compounds revealed that they have members of fungal G*α* subgroups I, II, and III (Figure 1). Rocha-Ramírez et al. [19] silenced and overexpressed *tga1*, encoding the subgroup I G*α* subunit in *T. atroviride* strain IMI 206040. Silencing of *tga1* led to intense sporulation and slowly growing colonies whereas overexpression had the opposite effect by promoting vegetative proliferation and increased coiling, a morphological change associated with the mycoparasitic host attack. In direct plate confrontation assays with *R. solani* as the host fungus, the transformed lines overexpressing *tga1* showed an impressive increase in the capacity of the fungus to overgrow and parasitize the host compared to the parental strain. On the other hand, lines blocked in the production of Tga1 were unable to overgrow the host [19].

A more profound functional characterization of Tga1 was performed by Reithner et al. [21], who extended the involvement of this G-protein *α* subunit to the production of antifungal metabolites and the formation of extracellular chitinases both processes relevant for the mycoparasitic host attack.* tga1* knockout mutants showed strongly reduced extracellular chitinase activities and a decreased transcription of the chitinase-encoding genes* nag1* (N-acetyl-glucosaminidase-encoding) and *ech42* (endochitinase 42-encoding). Investigation of the antifungal activity of the Δ*tga1* mutant revealed reduced amounts of the major antifungal metabolite of *T. atroviride*, 6-pentyl-*α*-pyrone [21], while elevated amounts of peptaibols, peptides with antibiotic activity, could be detected [60]. These results indicate contrasting functions of Tga1 in regulating the biosynthesis of different antifungal metabolites. An elevated internal steady-state cAMP level in the Δ*tga1* mutants compared to the parental strain confirmed that Tga1 represents a member of the adenylyl cylcase inhibiting subgroup I of fungal G*α* subunits [21]. 

Contrary to *T. atroviride* Tga1, its homologue TgaA does not influence growth or conidiation in *T. virens*, another mycoparasitic *Trichoderma* species. In antagonistic assays, when *Trichoderma* is confronted with a host fungus in a dual plate culture, *T. virens *Δ*tgaA* mutants showed a host-specific behavior as they could hardly colonise sclerotia of the plant pathogenic fungus* S. rolfsii* whereas they were fully pathogenic against another plant pathogen, *R. solani*. *T. virens *Δ*tgaB* mutants missing the subgroup II G*α* protein revealed unaltered growth, sporulation, and mycoparasitism of *R. solani* and sclerotia of *S. sclerotiorum* [20].

Functional characterization of the subgroup III G*α* protein Tga3 of *T. atroviride* revealed its involvement in regulating vegetative growth and conidiation. Δ*tga3 *knockout mutants exhibited significantly reduced intracellular cAMP levels compared to the parental strain [22]. Accordingly, examination of a *gna3*QL mutant of the only weakly mycoparasitic species *T. reesei*, carrying a constitutively activated allele of the subgroup III G*α* protein-encoding *tga3* homologue *gna3*, revealed a severe increase in intracellular cAMP levels [23, 61]. This confirmed the stimulatory role of the subgroup III G*α* proteins Tga3 and Gna3 on the activity of adenylyl cyclase. 

Analysis of the mycoparasitic activity of *T. atroviride *Δ*tga3* mutants in antagonistic plate assays revealed that they were completely avirulent, that is, they lost the ability to attack and lyse host fungi [22]. Microscopic characterization showed that the mutants were unable to form mycoparasitism-related infection structures, like attachment to and coiling around the host hyphae. Interestingly, addition of 5 mM exogenous cAMP to the confrontation assays led to a restoration of infection structure formation. When analyzing the production of cell wall lytic enzymes in Δ*tga3* knockout mutants, it turned out that Tga3 is also involved in regulating this mycoparasitism-relevant process. The mutants exhibited reduced levels of extracellularly secreted chitinases compared to the parental strain although they showed elevated transcription of the chitinase-encoding genes *nag1* and *ech42*. Further experiments revealed that chitinolytic enzymes are retained inside the cell suggesting an influence of Tga3 on chitinase gene transcription and secretion [22]. In *gna3*QL mutants of *T. reesei* elevated levels of different extracellular enzymes like endochitinase, N-acetyl-glucosaminidase, *β*-1,3-glucanase, lipase, and phosphatase were found [23]. In addition, the *gna3*QL mutants exhibited a significantly increased transcript abundance of the major cellulase-encoding gene *cbh1* compared to the parental strain when the fungus was cultivated in the presence of light [61]. The authors attribute this raise in enzyme production to the elevated intracellular cAMP levels caused by the constitutively activated Gna3 protein.

In addition to regulating infection structure formation and the production of cell-wall-degrading enzymes such as chitinases, *T. atroviride* Tga3 was also found to be required for the production of antifungal metabolites [22]. While there is a clear correlation between sporulation of the fungus and the secretion of antifungal metabolites in the *T. atroviride* parental strain, Δ*tga3 *mutants were fully impaired in the production of peptaibols although they exhibited a hypersporulating phenotype [62].

Interestingly, the Tmk1 MAP kinase was found to regulate the expression of chitinase-encoding genes in *T. atroviride* in a way similar to the Tga3 G*α* protein [63]. This suggests that a MAPK cascade involving Tmk1 acts downstream of Tga3 in governing chitinase production.

The crucial roles of the subgroup I and III G*α* proteins in regulating mycoparasitism-relevant processes implicates that identifying and clarifying the role of the corresponding G-protein-coupled receptors will be a fundamental step toward understanding the processes of host recognition and activation of the attack of phytopathogenic host fungi by mycoparasitic *Trichoderma* species. 

Recently, analysis of GPCRs in *Trichoderma* started and resulted in the *in silico* identification of more than 50 such receptors in the genome of both *T. reesei* [64] as well as the mycoparasitic species *T. atroviride* and *T. virens* (S. Zeilinger, M. Omann, unpublished). Based on this analysis, four GPCR-encoding genes from the mycoparasite *T. atroviride* were isolated and further characterized. The obtained results showed that at least the Gpr1 receptor, grouping to the class of cAMP receptor-like (CRL) proteins (class V of fungal GPCRs), plays a major role during vegetative growth and conidiation in *T. atroviride* [64].

Furthermore, mutants bearing a *gpr1* gene whose expression is silenced by an RNAi approach, showed a complete loss of mycoparasitism accompanied by a failure to attach to and coil around host hyphae. Interestingly, this defect in host recognition and infection structure formation could be restored by addition of exogenous cAMP [65]—similar to what was found for *T. atroviride *Δ*tga3* mutants [22]. These results suggest that Gpr1 regulates infection structure formation via the cAMP-pathway by signaling via the Tga3 G*α* protein.

## 4. Conclusions

Mycoparasitism comprises the interaction between two fungi involving an elaborate cross-talk of the host and the pathogen. During recent years, an increasing number of studies on the signaling pathways participating in this interaction have been performed and revealed high conservation of the investigated compounds from mycoparasitic *Trichoderma* to homologous proteins from other fungi.

Accordingly, signaling pathways employing, for example, G*α* subunits of heterotrimeric G proteins, mitogen-activated protein kinases, adenylyl cyclases, and G-protein-coupled receptors have been shown to be important for virulence in fungi being pathogenic to plants animals/humans, as well as mycoparasites.

**Figure 2:**
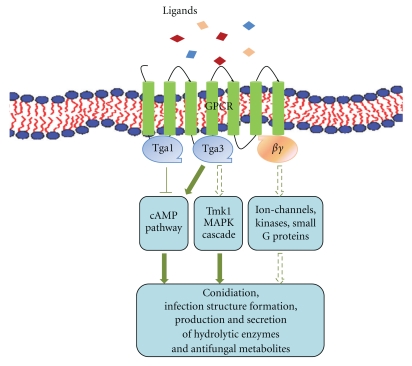
Schematic illustration of G-protein signaling in *Trichoderma atroviride. *GPCR: G protein-coupled receptor; Tga1, Tga3: subgroup I and III G-protein *α* subunits; Tmk1: MAP kinase.

In mycoparasitic *Trichoderma *species, both subgroup I and subgroup III G*α* proteins were shown to govern mycoparasitism-relevant processes such as the production of cell wall lytic enzymes and antifungal metabolites and the formation of infection structures. Both subgroup I and III G*α* proteins of *T. atroviride* signal—at least partially—via the cAMP pathway as Tga1 was proven to negatively influence the activity of adenylate cyclase whereas Tga3 stimulated its activity (Figure 2). 

The essential role of G-protein signaling in activation of the mycoparasitic response of *Trichoderma* was further supported by the functional characterization of *T. atroviride* Gpr1. Similar to the Tga3 G*α* subunit, the Gpr1 G-protein-coupled receptor seems to be involved in recognizing host-derived signals and transducing them via the cAMP pathway. Gpr1, therefore, is the first GPCR from a mycoparasitic fungus which was functionally characterized and the first 7-transmembrane receptor belonging to the CRL class of fungal GPCRs for which pathogenicity-related functions could be shown. 

Although during recent years there has been considerable progress in elucidating G-protein-mediated signaling in pathogenic fungi, there are still many unsolved questions especially concerning host recognition and regulation of signaling events. The elucidation of these processes will for sure be highly beneficial not only for developing mechanisms and substances for combating these pathogens, but also for a better understanding of the molecular processes underlying fungal mycoparasitism.
